# Antitumor, Antioxidant, and Hepatoprotective Effects of Grape Seed Oil Nanoemulsion as a Dietary Phytochemical Intervention in Ehrlich Solid Tumors

**DOI:** 10.3390/nu17213450

**Published:** 2025-10-31

**Authors:** Aly A. M. Shaalan, Ekramy M. Elmorsy, Eman M. Embaby, M. Alfawaz, Nagwa M. Aly, Ahmed S. Shams, Manal S. Fawzy, Nora Hosny

**Affiliations:** 1 Department of Anatomy, Faculty of Medicine, Jazan University, Jazan 45142, Saudi Arabia; ashaalan@jazanu.edu.sa; 2 Department of Histology and Cell Biology, Faculty of Medicine, Suez Canal University, Ismailia 41522, Egypt; 3 Center for Health Research, Northern Border University, Arar 73213, Saudi Arabia; ekramy.elmorsy@nbu.edu.sa; 4 Department of Forensic Medicine and Clinical Toxicology, Faculty of Medicine, Mansoura University, Mansoura 35516, Egypt; 5 Department of Physiology, Faculty of Veterinary Medicine, Mansoura University, Mansoura 35516, Egypt; emanmohamed@mans.edu.eg; 6 Department of Medical Laboratory Technology, College of Applied Medical Sciences, Northern Border University, Arar 91431, Saudi Arabia; mohammed.alfawaz@nbu.edu.sa; 7 Department of Medical Biochemistry and Molecular Biology, Faculty of Medicine, Suez Canal University, Ismailia 41522, Egypt; nagwaaly@med.suez.edu.eg (N.M.A.); nora_hosny@med.suez.edu.eg (N.H.); 8 Department of Human Anatomy and Embryology, Faculty of Medicine, Suez Canal University, Ismailia 41522, Egypt; ashams@umn.edu

**Keywords:** grape seed oil, nanoemulsion, Ehrlich solid tumor, antioxidant activity, antitumor activity

## Abstract

**Background/Objectives:** Grape seed oil (GSO) is a potent source of dietary phytochemicals, particularly polyphenols and flavonoids, known for their health-promoting properties. This study aims to investigate the anticancer and hepatoprotective effects of a nanoemulsion formulation of grape seed oil (GSONE), to enhance the efficacy and bioavailability of its phytochemical constituents against solid tumors. **Methods:** Ninety female Swiss albino mice were divided into six groups: control, alone, GSONE alone, Ehrlich solid tumor (EST), EST treated with GSO, and EST treated with GSONE. Tumor development, growth performance, serum biochemistry, antioxidant status, hepatic histopathology, apoptotic gene expression, and flow cytometry analyses were assessed following 30 days of daily oral treatment. **Results:** GSONE significantly reduced tumor weight and volume (52.9%) and more effectively counteracted tumor-induced body weight loss than crude GSO. Treatment with GSONE normalized serum protein levels and improved liver function markers (AST, ALT, ALP, total bilirubin) to near-control values. Tumor markers (AFP, CEA) and oxidative stress indices (MDA, 8-OHdG) were markedly decreased, while activities of hepatic antioxidants (SOD, CAT, GPx, GSH) were restored. GSONE enhanced gene expression of pro-apoptotic markers (Bax, TP53, caspase-3, caspase-9), suppressed anti-apoptotic Bcl-2, and significantly increased the proportion of p53- and cleaved caspase-3-positive tumor cells. Liver histopathology and ultrastructure demonstrated normalized morphology and reduced damage in GSONE-treated mice. Multivariate analyses confirmed GSONE’s restorative effect compared to raw GSO. **Conclusions:** The delivery of dietary phytochemicals via nanoemulsion significantly enhances antitumor and hepatoprotective actions in a preclinical solid tumor model. These findings support the potential of phytochemical-rich edible oils, enhanced by nanotechnology, for dietary prevention and adjunctive management of cancer.

## 1. Introduction

Dietary phytochemicals, naturally occurring bioactive compounds in plant-derived foods, have gained increasing recognition for their role in cancer chemoprevention and therapy [1]. Polyphenols, flavonoids, and related compounds modulate redox homeostasis, regulate apoptosis, and influence multiple molecular pathways involved in carcinogenesis [2]. Accumulating evidence supports that phytochemical-rich diets may lower cancer risk and improve disease outcomes [3]. Recent advances in nutrition and pharmaceutical technology have underscored the importance of optimizing the delivery, stability, and bioavailability of these compounds to maximize their therapeutic impact [4,5].

Grape seeds and their extracted oil are valuable agricultural by-products of grape processing, recognized for their high nutritional value and rich phytochemical composition [6]. Grape seed oil (GSO) is obtained mainly by cold pressing or solvent extraction of Vitis vinifera seeds, which contain approximately 8–20% oil. This oil is particularly valued for its delicate flavor, high smoke point, and balanced lipid composition, making it useful for both culinary and therapeutic applications [7,8].

Nutritionally, grape seed oil is rich in polyunsaturated fatty acids (PUFAs), which account for nearly 85–90% of its fatty acid content. Linoleic acid (C18:2, *n*-6) constitutes around 68–76%, while oleic acid (C18:1, *n*-9) represents 14–15%, accompanied by small fractions of alpha-linolenic, palmitic, and stearic acids [9]. This lipid profile contributes to maintaining cardiovascular and metabolic health by promoting favorable lipid metabolism and supporting membrane fluidity [7]. GSO is also an exceptional natural source of vitamin E (1–53 mg/100 g), mainly in the form of γ-tocopherol and tocotrienols, compounds known for their potent antioxidant and anti-aging properties [10].

**Figure 1:**
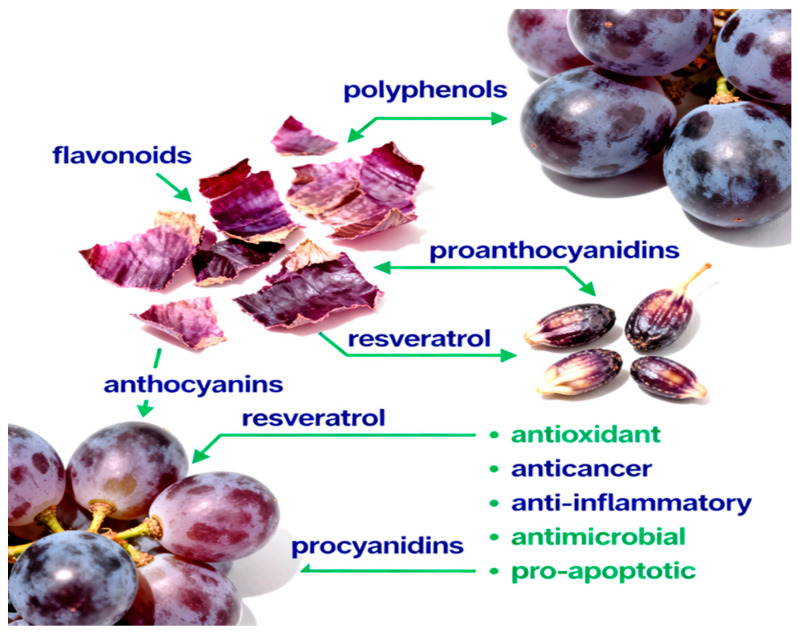
The phytochemical composition of grape skins and seeds includes polyphenols, flavonoids, proanthocyanidins, anthocyanins, resveratrol, and procyanidins. These compounds exhibit diverse biological activities, including antioxidant, anticancer, anti-inflammatory, antimicrobial, and pro-apoptotic effects, which contribute to their potential to promote health and prevent disease.

In addition to lipophilic antioxidants, grape seed oil contains trace hydrophilic bioactives such as polyphenols (catechins, epicatechins, procyanidins, and resveratrol derivatives) and phytosterols (β-sitosterol, campesterol, and stigmasterol) [11]. These components contribute synergistically to its broad spectrum of biological activities, including antioxidant, anti-inflammatory, anti-obesity, hepatoprotective, and antitumor effects demonstrated in both in vitro and in vivo models [12,13]. Figure 1 provides an overview of the main classes of bioactive compounds present in grape skins and seeds, and summarizes their documented health-promoting actions.

Beyond its dietary importance, grape seed oil has been incorporated into several pharmaceutical and cosmeceutical formulations, including (a) nanoemulsions and liposomal carriers, which enhance solubility, stability, and absorption of natural antioxidants [14,15], and (b) topical and oral nutraceutical preparations for skin protection, wound healing, lipid regulation, and systemic antioxidant support [16,17,18]. These formulations aim to overcome the poor aqueous solubility and oxidative instability associated with the native oil, thereby improving its therapeutic performance [19,20].

Despite GSO’s diverse health-promoting constituents, its high *n*-6 PUFA content predisposes it to oxidation, limiting its direct storage and industrial use [21]. Therefore, nanoformulation approaches, such as emulsions and encapsulated systems, have attracted substantial attention for enhancing bioavailability and shelf stability [20,22,23]. In this context, grape seed oil nanoemulsion (GSONE) represents a novel strategy to potentiate the therapeutic effects of dietary phytochemicals while maintaining biocompatibility and safety [24].

Experimental cancer models remain indispensable for preclinical testing of such nutritional interventions. The Ehrlich solid tumor (EST) model, derived from murine mammary adenocarcinoma, closely mimics the clinical and histopathological features of aggressive human breast tumors, such as rapid proliferation, high malignancy, short biological cycles, and lack of tumor-specific transplantation antigens [25]. Both solid and ascitic forms of Ehrlich carcinoma are routinely used to investigate tumor biology and assess novel antitumor strategies [26,27,28]. Tumor progression in the EST model is known to trigger systemic metabolic imbalances and organ dysfunction, notably hepatic and renal oxidative stress, DNA damage, and cellular injury [29]. Such alterations highlight the pivotal role of reactive oxygen species (ROS) in carcinogenic processes and reinforce the protective role of natural antioxidants in maintaining redox balance and genomic stability [30,31]. Dietary antioxidants thus represent a physiologically relevant and ethically sustainable avenue for developing biocompatible cancer-preventive adjuvants [32].

The present study aimed to develop a GSONE to overcome the intrinsic limitations of native GSO and to investigate its chemopreventive, antitumor, and hepatoprotective properties in a murine EST model. We further sought to elucidate the underlying molecular mechanisms by assessing apoptosis-related gene expression, hepatic redox indices, and histopathological alterations following chronic treatment.

## 2. Materials and Methods

### 2.1. Preparation of Grape Seed Oil Nanoemulsion

Organic grape seed oil (GSO) was obtained from AB Chem Company (Mansoura City, Egypt). A monolayer nanoemulsion was formulated using 20% GSO. Briefly, the oil phase was gradually mixed with 68 mL of an aqueous phase containing 10% Tween 80 (surfactant) and 2% ethanol (co-surfactant) under gentle stirring at 25 °C. Water was added at a controlled rate of 1.0 mL/min to maintain uniform emulsification. The emulsion was subjected to ultrasonic bath treatment for 30 min, followed by final homogenization with a probe-type ultrasonic homogenizer (Sonics Vibra-Cell, VC 505, Newtown, CT, USA) at 60% amplitude for 5 min, with a one-second on and one-second off cycle. This process yielded a stable GSO nanoemulsion (GSONE).

For morphological analysis, freshly prepared GSONE samples were visualized using a 160 kV transmission electron microscope (JEOL JEM-2100, Akishima, Japan). Image acquisition and processing were performed with Soft Imaging Viewer and Digital Micrograph (Gatan Microscopy Suite, version 2.11.1404.0). Particle size distribution (Z-average), polydispersity index (PDI), and zeta potential were determined using a Zeta sizer Nano ZS analyzer (Malvern Instruments, Malvern, UK).

### 2.2. Induction of EAC in Mice

The Ehrlich Ascites Carcinoma (EAC) cell line was obtained from the National Cancer Institute (NCI), Cairo, Egypt. To preserve cell viability, EAC cells were serially passaged in vivo by intraperitoneal injection of 2.5 × 10^6^ cells into female Swiss albino mice every 10 days, following the protocol described by Yılmaz et al. [33].

Only female mice were selected for this study because the EAC model originates from murine mammary tissue and most closely simulates the biological behavior, treatment response, and progression patterns of breast cancer seen in women. Utilizing females not only reflects the clinical distribution of breast cancer but also conforms to standard EAC experimental practices, thereby enhancing the translational relevance and scientific rigor of antitumor investigations [25,34].

Mice were anesthetized with intraperitoneal administration of ketamine (80 mg/kg) and xylazine (10 mg/kg). Ascitic fluid was collected from EAC-bearing donor mice on days 7 or 8 post-inoculation under identical anesthesia conditions. Cell viability was assessed using a hemocytometer and 0.4% trypan blue dye exclusion, as described previously [35]. The viable cell suspension was then adjusted to 2.5 × 10^6^ cells in 0.2 mL phosphate-buffered saline (PBS) for subsequent inoculation into experimental mice.

### 2.3. Animal Handling and Experimental Protocol

**Figure 2:**
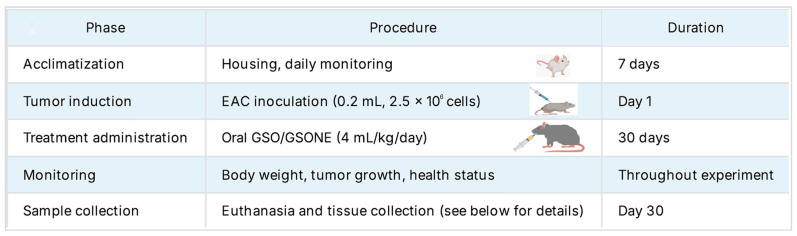
The overall study timeline and protocol were followed in this experiment.

A total of ninety healthy female Swiss albino mice (*Mus musculus*; mean body weight: 21.61 g) were provided by the Faculty of Medicine, Mansoura University, Egypt. Animals were housed in plastic cages under controlled laboratory conditions (25 °C, 45% relative humidity, 12 h light/dark cycle) with ad libitum access to food and water. Mice were allowed to acclimate for one week under these standardized conditions. The overall experiment timeline is summarized in Figure 2.

All animal care and procedures were conducted in accordance with the “Guide for the Care and Use of Laboratory Animals (8th edition, NRC, 2011) and the International Guiding Principles for Biomedical Research Involving Animals (1985).” All animal procedures were reviewed and approved by the “Research Ethics Committee of the Faculty of Veterinary Medicine, Mansoura University, Egypt (approval code: MU-ACUC; VM.R.25.10.247).”

A preliminary pilot study was conducted to assess the safety margin of grape seed oil (GSO) in healthy mice, with oral doses of 1, 2, 3, and 4 mL/kg/day administered for 30 consecutive days. The upper dose limit was selected based on previous reports of hepatoprotective efficacy in mice [36]. Throughout, animals were monitored for mortality, behavior, and toxicity. As no adverse effects were observed at 4 mL/kg/day, this dose was used for all subsequent experiments.

Mice were randomly allocated into six groups (*n* = 15 each):

- Group 1 (Control): received no treatment.
- Group 2 (GSO): received GSO orally at 4 mL/kg/day for 30 days.
- Group 3 (GSONE): received GSO nanoemulsion (GSONE) orally at the same dose.
- Group 4 (EST): inoculated intramuscularly with 0.2 mL of EAC cell suspension (2.5 × 10^6^ cells) in the right thigh on day 1 and left untreated thereafter.
- Group 5 (EST + GSO): received daily oral GSO (4 mL/kg) for 30 days following EAC inoculation.
- Group 6 (EST + GSONE): received daily oral GSONE (4 mL/kg) for 30 days following EAC inoculation.

The sample size for each group was determined with reference to previous EAC murine studies, in which group sizes of 10–12 animals were found sufficient to detect biological and tumor-related differences. To achieve a statistical power of at least 80% for pairwise group comparisons at a significance level of α = 0.05, and to accommodate potential attrition due to post-inoculation mortality (anticipated at 10–20%), the group size was set at 15. This design provides adequate power for one-way ANOVA analysis across six groups (total *n* = 90), aligning with established international standards for rigor and ethical animal use in preclinical research [37,38]. No specific pre-established inclusion or exclusion criteria were applied in this study; all animals enrolled in the study completed the experimental protocol.

### 2.4. Assessment of Body Weight, Tumor Weight, and Tumor Volume

Body weights were recorded for all animals at baseline (day 0) and at the conclusion of the experimental period. Net final body weight was determined by subtracting the excised tumor mass from the final body weight. Tumor volume was measured at regular intervals from day 8 to day 30 in the EST, EST + GSO, and EST + GSONE groups using digital Vernier calipers. Tumor dimensions (major axis, B; minor axis, A) were used to calculate volume according to the formula: TV (mm^3^) = 0.52 × A × B^2^ [39]. On day 30, animals were euthanized, and tumors were excised and weighed to determine the final tumor weight.

### 2.5. Sample Collection

At the end of the experimental period, 10 randomly selected mice from each group were fasted for 10 h and anesthetized with inhaled tetrahydrofuran to minimize distress and ensure consistent blood collection. The remaining mice (*n* = 5 for each group) served as contingency reserves in case of mortality, unavailable samples, or insufficient tissue/RNA yields. Animals were euthanized by cervical dislocation. Blood was collected from the retro-orbital venous sinus into non-heparinized tubes, allowed to clot at room temperature, and centrifuged at 3000× *g* for 10 min. Serum was separated and immediately stored in 0.5 mL Eppendorf tubes for subsequent biochemical analyses.

### 2.6. Serum Biochemical Analysis

Serum biochemical parameters were measured using commercially available diagnostic kits, according to the manufacturers’ protocols. Analytes included total protein (TP; CBP007-K), albumin (MET-5017), total bilirubin (TB; BR 1001), alkaline phosphatase (ALP; CBA-301), alanine aminotransferase (ALT; MET-5123), and aspartate aminotransferase (AST; MET-5127); globulin was calculated as the difference between total protein and albumin. Alpha-fetoprotein (AFP) was quantified using the mini-VIDAS^®^ automated ELFA system (Biomerieux, Marcy-L’Étoile, France), while carcinoembryonic antigen (CEA) was measured with a Mouse CEA ELISA Kit (MyBioSource, San Diego, CA, USA), both per manufacturers’ instructions.

### 2.7. Assessment of Antioxidant Status and DNA Oxidative Damage

Liver samples were washed in 1.15% ice-cold potassium chloride to remove blood residue. Specimens destined for biochemical assays were rapidly frozen at −20 °C, and others were fixed in 10% neutral-buffered formalin for microscopy. Homogenization was performed in ice-cold phosphate buffer (pH 7.4), followed by centrifugation at 4000 rpm (4 °C, 15 min). Supernatants were used to determine activities of superoxide dismutase (SOD; Cat. No. SD 2521), catalase (CAT; CA 2517), glutathione peroxidase (GSH-Px; GP 2524), and reduced glutathione (GSH; GR 2511). Lipid peroxidation was measured as malondialdehyde (MDA) by TBARS assay (MDA; MD 2529). DNA oxidative damage was assessed by quantifying 8-hydroxy-2′-deoxyguanosine (8-OHdG) using a competitive ELISA kit (Trevigen, Gaithersburg, MD, USA). Absorbance was measured at 450 nm, and results were calculated from a standard curve, expressed as nanograms per milligram of tissue protein.

### 2.8. Gene Expression Analysis of Apoptotic Markers

Total RNA was extracted from tumor tissues of mice bearing EST using the Gene JET™ RNA Purification Kit (Thermo Scientific, Waltham, MA, USA) according to the manufacturer’s protocol. RNA concentration and purity were quantified by spectrophotometry (NanoDrop, Wilmington, DE, USA), and integrity was confirmed by agarose gel electrophoresis, which showed distinct 28S and 18S rRNA bands with a 2:1 intensity ratio. First-strand complementary DNA (cDNA) was synthesized from 5 µg of total RNA using the RevertAid First Strand cDNA Synthesis Kit (Thermo Scientific). Gene-specific primers for *Bax*, *Bcl-2*, *TP53*, *Caspase-3*, *Caspase-9*, and the housekeeping gene Glyceraldehyde phosphate dehydrogenase (*GAPDH*) (Table 1) were designed with NCBI Primer-BLAST and synthesized by Invitrogen (Carlsbad, CA, USA). *GAPDH* served as the internal control for normalization. Primer sequences were based on methodologies reported by Mohamed et al. [40] and Gencer et al. [41].

### 2.9. Flow Cytometric Analysis of p53 and Caspase-3 Expression

Single-cell suspensions were prepared from EST of control and treated mice (GSO, GSONE) to assess apoptotic pathway activation by quantifying Tp53 and cleaved caspase-3 expression. Excised tumor tissues were minced under sterile conditions, enzymatically dissociated in 1 mg/mL collagenase type I (Sigma-Aldrich, St. Louis, MO, USA) in PBS (pH 7.4) at 37 °C with gentle agitation for 30 min, and then filtered through a 70 µm nylon mesh. Cells were washed twice in ice-cold PBS (centrifuged at 300× *g* for 5 min), then fixed and permeabilized using the Cytofix/Cytoperm kit (BD Biosciences, Franklin Lakes, NJ, USA). For p53 detection, permeabilized cells were incubated with an Alexa Fluor 488-conjugated anti-p53 antibody (Abcam, ab225239) according to the manufacturer’s protocol. Cleaved caspase-3 was detected with a phycoerythrin (PE)-conjugated anti-cleaved caspase-3 antibody (Abcam, ab32042).

To ensure analytical rigor and reproducibility, cell viability was monitored throughout the sample preparation process. Appropriate controls, including unstained, isotype-matched, and single-color compensation controls, were used to gate the data and confirm antibody specificity accurately. Instrument calibration was performed daily using standardized fluorescent beads, and acquisition and compensation settings were standardized for all samples. Each analysis included at least 10,000 single, viable cell events, and doublet discrimination was routinely applied during gating. Data were collected on a BD FACSCanto™ II flow cytometer (BD Biosciences) and analyzed with FlowJo™ software (version 10.8.1). Mean fluorescence intensity (MFI) and the percentage of positively stained cells were determined for each marker, enabling comparisons among the study groups.

### 2.10. Histopathological Analysis

After fixation in 10% neutral-buffered formalin, liver tissues were dehydrated using a graded ethanol series (70%, 80%, 90%, and 100%, one hour per step), then cleared in two successive xylene baths before paraffin embedding. Specimen identity was carefully maintained throughout processing, and all samples were checked at receipt for correct labeling and presence of adequate fixative to avoid pre-analytic errors. Paraffin blocks were sectioned using a rotary microtome at an approximate thickness of 5 µm. Serial sections were mounted, stained with hematoxylin and eosin (H&E), and examined microscopically. Each batch of staining included an established normal liver tissue section as an internal control to ensure consistency of stain quality and interpretation.

Microscopic evaluation was performed using a high-resolution digital camera to document representative fields of interest. For semi-quantitative assessment, liver lesions were graded in three mice per group, analyzing three sections per slide and four randomly selected fields per section at 400× magnification (totaling 12 fields per animal). All fields were scored independently by a blinded observer using a standardized four-grade histopathological scoring system (0 = none, 1 = mild, 2 = moderate, 3 = severe; see Table 2), and the mean lesion score for each mouse was calculated by averaging across all examined fields. Quality of tissue sections and staining was monitored throughout, and any slide with a technical artifact or inadequate staining was reprocessed and reassessed to maintain high analytical standards.

### 2.11. Transmission Electron Microscopy (TEM)

Liver samples were fixed in 2.5% glutaraldehyde (0.1 M phosphate buffer, pH 7.4) at 4 °C for 24 h to preserve their ultrastructure. Following thorough rinsing, specimens were post-fixed in 1% osmium tetroxide for 2 h. Tissues were dehydrated through a graded ethanol series (50–100%) and cleared in acetone before being embedded in epoxy resin. Ultrathin sections (60–70 nm) were cut using a diamond knife on an ultramicrotome. Sections were mounted on copper grids, stained with uranyl acetate and lead citrate, and examined with a JEOL 2100 transmission electron microscope (Japan) operated at 160 kV. Each processing batch included internal controls, and section quality was confirmed before imaging. Representative regions were selected for examinations, and digital images were acquired to minimize operator bias.

### 2.12. Statistical Analysis

Before analysis, the normality of data distribution and homogeneity of variances were verified using the Shapiro–Wilk and Levene’s tests, respectively. One-way ANOVA (SAS Institute, 2012, Proc ANOVA) was performed to compare means among experimental groups, and Tukey’s post hoc test was employed for multiple comparisons. Results are reported as mean ± standard error (SE), with statistical significance set at *p* < 0.05. Data visualization was performed with GraphPad Prism 9.0, and multivariate analyses (principal component analysis (PCA) and heatmap clustering) were conducted with SRplot.

## 3. Results

### 3.1. Characterization of Grape Seed Oil Nanoemulsion (GSONE)

**Figure 3:**
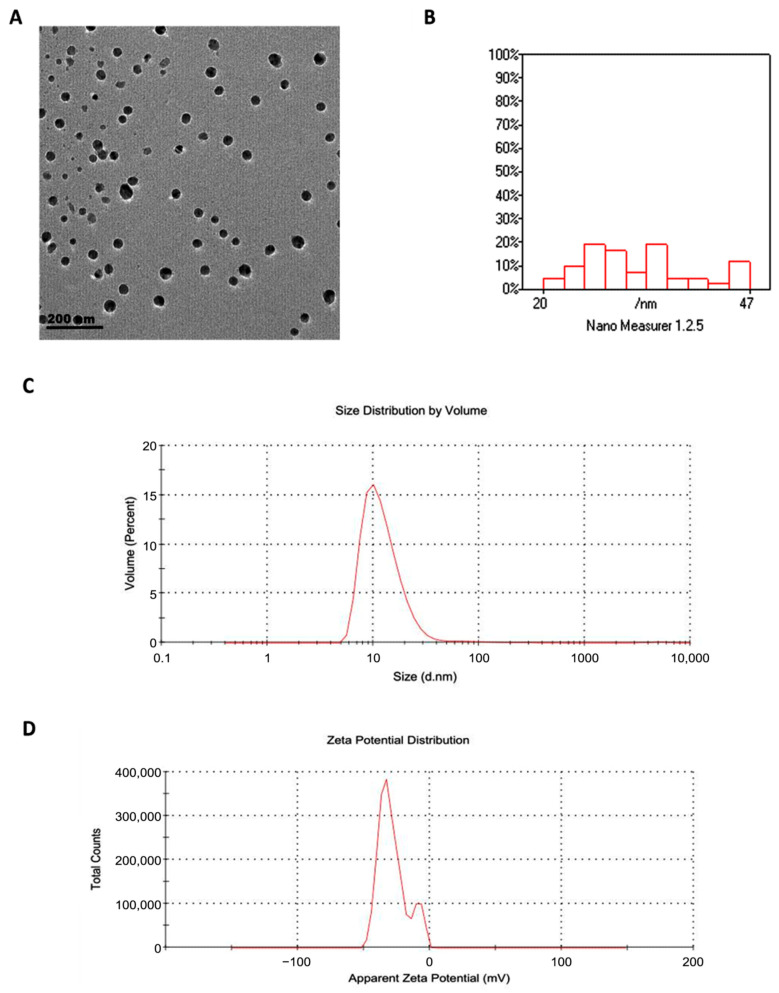
Physicochemical characterization of grape seed oil nanoemulsion (GSONE). (**A**) Transmission electron microscopy (TEM) image shows predominantly spherical and uniformly dispersed nanoparticles. (**B**) Particle size distribution histogram indicates that most particles range from 20 to 47 nm in diameter. (**C**) Dynamic light scattering (DLS) analysis displays size distribution by volume (Z-average: 88 nm; PDI: 0.542). (**D**) Zeta potential measurement reveals a value of −28 mV, indicative of electrostatic stability of the nanoemulsion suspension.

Transmission electron microscopy revealed that GSONE particles were spherical and uniform in morphology (Figure 3A) and ranged in diameter from 20 to 47 nm (Figure 3B). Dynamic light scattering (DLS) analysis yielded a hydrodynamic diameter of 88 nm, which is larger than the TEM values, reflecting particle-solvent interactions and hydration shell formation. The PDI was 0.542, suggesting moderate size dispersion within the formulation (Figure 3C). The zeta potential was measured at −28 mV, confirming good electrostatic stability of the nanoemulsion suspension (Figure 3D). All measurements were performed on freshly prepared samples and independently verified in replicate runs to ensure consistency and accuracy.

### 3.2. Growth Performance and Tumor Weight/Volume Changes

**Figure 4:**
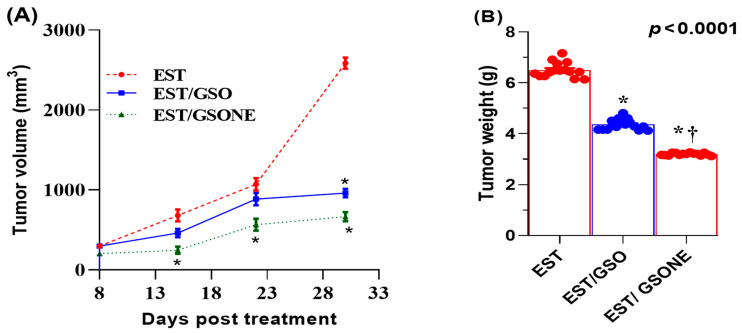
Changes in Tumor volume (**A**) and tumor weight (**B**) in mice bearing Ehrlich solid carcinoma (EST) and treated with grape seed oil nanoemulsion or raw grape seed oil. Each group (*n* =15). EST: Inoculated with Ehrlich Ascites Carcinoma (EAC) cells (0.2 mL); EST/GSO: Grape seed oil (4 mg/kg body weight) + EAC cells (0.2 mL); EST/GSONE: Grape seed oil nanoemulsion (4 mg/kg body weight) + EAC cells (0.2 mL) (each group *n* = 15. Standard bars represent the standard error of the mean (SEM). * Significantly different from the EST group; † Significantly different from the EST/GSO group.

Treatment effects on growth performance and tumor burden were quantified in mice bearing EST following administration of either crude GSO or its nanoemulsion form (Table 3). The EST group displayed a significant reduction in net body weight relative to all controls. Both GSO and GSONE therapies improved body weight, with the EST/GSONE group demonstrating a greater reversal of cachexia than the EST/GSO group. Tumor weight was highest in the untreated EST group, while both treatments led to marked reductions; notably, GSONE yielded the most significant effect, achieving a 52.89% decrease in tumor mass compared to the untreated EST group. Across all measurement time points, tumor volume was lowest in the EST/GSONE group, intermediate in the EST/GSO group, and highest in the untreated EST group, with nanoemulsion consistently outperforming crude oil (Figure 4).

### 3.3. Tumor Histopathology

**Figure 5:**
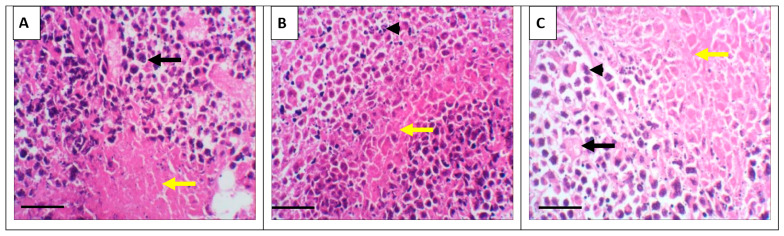
Representative photomicrograph of solid tumors from the positive control and different experimental groups. (**A**) The EAC group shows tumor tissue infiltrating the muscle layer and forming solid sheets of undifferentiated tumor cells (yellow arrow), with tumor cells showing high-grade anaplasia, pleomorphism, and abnormal mitosis (black arrow). (**B**) The treated group with grape seed oil (GSO) showed a massive reduction in tumour size and cell count (arrowhead) between the skeletal muscle bundle (yellow arrow). (**C**) The treated group with GSO nanoemulsion (GSONE) showed a reduction in the quantity of the active neoplastic cells, vacuolation (black arrows), and degeneration of the neoplastic cells with karyolysed nuclei (arrowhead) and a skeletal muscle bundle (yellow arrow). All images at = 400×. Scale bar = 50 µm.

Representative hematoxylin and eosin (H&E)-stained sections of tumor tissues from each experimental group are shown in Figure 5. Distinct histopathological features were evident among the groups. The control group displayed uniform, densely packed tumor cells with occasional necrotic foci. In contrast, treatment with GSO (Panel B) and, particularly, GSONE (Panel C) resulted in marked changes in tumor architecture, including reduced cellular density, prominent areas of necrosis, and greater stromal separation. These morphological changes underscore the modulatory effects of GSO and GSONE on tumor tissue structure and are consistent with reduced tumor aggressiveness and enhanced therapeutic response.

### 3.4. Blood Biochemical Parameters

Blood biochemical profiling revealed pronounced disruptions in protein metabolism and liver function in mice bearing EST (Table 4). The EST group showed significant reductions in serum total protein, albumin, and globulin levels compared with controls. Administration of GSONE significantly improved these parameters, normalizing values to levels indistinguishable from those of the control group. Globulin concentrations remained similar between the EST/GSO and EST/GSONE treated groups. Markers of hepatic injury, including AST, ALT, ALP, and total bilirubin, were significantly elevated in the EST group compared to controls. Both GSO and GSONE treatments significantly attenuated these elevations, with the most significant restorative effect observed in the EST/GSONE cohort, whose values approached those of healthy controls.

### 3.5. Tumor-Associated Biomarkers

**Figure 6:**
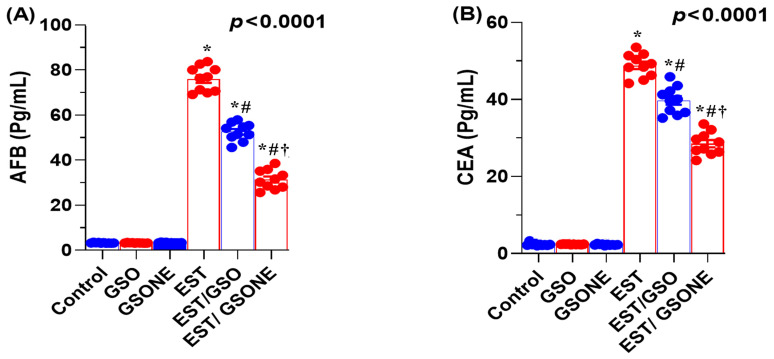
Effects of grape seed oil nanoemulsion (GSONE) and crude grape seed oil (GSO) on serum tumor-associated biomarkers in Ehrlich solid carcinoma (EST)-bearing mice. (**A**) Alpha-fetoprotein (AFP) and (**B**) carcinoembryonic antigen (CEA) serum levels in control, EST, EST/GSO, and EST/GSONE groups. GSO: Grape seed oil (4 mg/kg body weight); GSONE: Grape seed oil nanoemulsion (4 mg/kg body weight); EST: Inoculated with Ehrlich Ascites Carcinoma (EAC) cells (0.2 mL); EST/GSO: GSO + EAC cells; EST/GSONE: GSONE + EAC cells. Data are presented as mean ± SE (each group *n* = 10). Statistical significance was determined by one-way ANOVA with Tukey’s post hoc test. * Significantly different from the control group; # Significantly different from the EST group; † Significantly different from the EST/GSO group.

Serum concentrations of AFP and CEA were markedly elevated in mice bearing EST compared with all control groups, indicating a higher tumor burden. Upon treatment with crude GSO or its nanoemulsion counterpart (GSONE), both AFP and CEA levels were significantly reduced. Notably, the EST/GSONE group showed a more pronounced decrease in tumor markers than the EST/GSO group, supporting the enhanced antitumor efficacy of the nanoemulsion formulation (Figure 6A,B). These findings support the value of AFP and CEA as sensitive indicators of therapeutic response and tumor suppression in this model.

### 3.6. Redox Status and DNA Oxidative Damage

**Figure 7:**
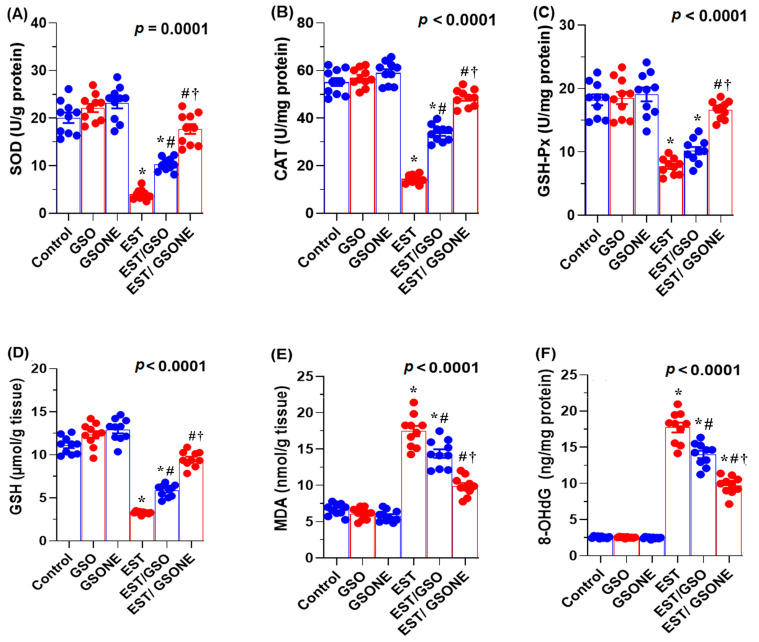
Effects of grape seed oil nanoemulsion (GSONE) and crude grape seed oil (GSO) on hepatic redox status and DNA oxidative damage in Ehrlich solid carcinoma (EST)-bearing mice. (**A**–**D**) Antioxidant enzyme activities: superoxide dismutase (SOD), catalase (CAT), glutathione peroxidase (GSH-Px), and reduced glutathione (GSH) levels; (**E**) hepatic malondialdehyde (MDA) concentrations as a marker of lipid peroxidation; (**F**) 8-hydroxy-2′-deoxyguanosine (8-OHdG) levels as an indicator of DNA oxidative damage. GSO: Grape seed oil (4 mg/kg body weight); GSONE: Grape seed oil nanoemulsion (4 mg/kg body weight); EST: Inoculated with Ehrlich Ascites Carcinoma (EAC) cells (0.2 mL); EST/GSO: GSO + EAC cells; EST/GSONE: GSONE + EAC cells. Each group (*n* = 10). Bars represent mean ± SE. A one-way ANOVA with Tukey’s post hoc test was used. * Significantly different from the control group; # Significantly different from the EST group; † Significantly different from the EST/GSO group.

Mice bearing EST exhibited significantly decreased hepatic antioxidant defenses, as evidenced by lower levels of reduced GSH, SOD, CAT, and GSH-Px compared to healthy controls. Treatment with either crude GSO or its nanoemulsion (GSONE) markedly restored these antioxidant parameters, with the EST/GSONE group demonstrating the most robust recovery across all markers (Figure 7A–D). Tumor-induced oxidative stress, as reflected by elevated MDA concentrations, was significantly reduced after intervention, with GSONE showing superior efficacy and restoring MDA levels to those of the control group (Figure 7E). Furthermore, DNA oxidative damage, assessed by hepatic 8-hydroxy-2′-deoxyguanosine (8-OHdG), was highest in the EST group and effectively minimized following treatment, with the lowest 8-OHdG values observed in the EST/GSONE cohort (Figure 7F). These data underscore the potent antioxidant and protective effects of GSONE against tumor-induced oxidative stress and genotoxicity.

### 3.7. Apoptotic Gene Expression Profiles

**Figure 8:**
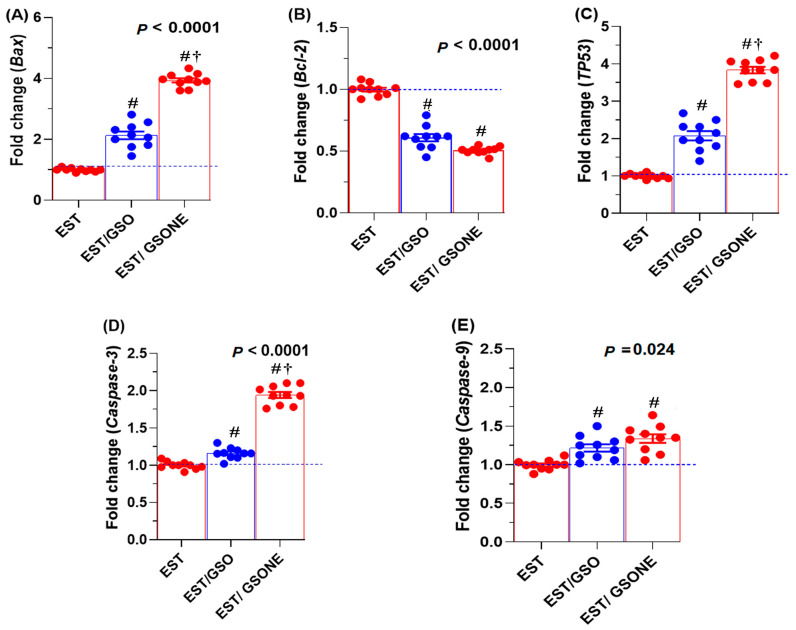
Effects of grape seed oil nanoemulsion (GSONE) and crude grape seed oil (GSO) on apoptotic gene expression profiles in Ehrlich solid carcinoma (EST)-bearing mice. Expression levels of (**A**) *Bax* (Bcl-2-associated X protein), (**B**) *Bcl-2* (B-cell lymphoma 2), (**C**) *TP53* (tumor protein 53), (**D**) *Caspase-3* (cysteine-aspartic acid protease-3), and (**E**) *Caspase-9* (cysteine-aspartic acid protease-9) in control, EST, EST/GSO, and EST/GSONE groups. GSO: Grape seed oil (4 mg/kg body weight); GSONE: Grape seed oil nanoemulsion (4 mg/kg body weight); EST: Inoculated with Ehrlich Ascites Carcinoma (EAC) cells (0.2 mL); EST/GSO: GSO + EAC cells; EST/GSONE: GSONE + EAC cells. Each group (*n* = 10). Values are presented as mean ± SE. Statistical comparisons were performed using one-way ANOVA with Tukey’s post hoc test. # Significantly different from the EST group; † Significantly different from the EST/GSO group.

Expression analysis revealed that pro-apoptotic genes, including *Bax*, *TP53*, *caspase-3*, and *caspase-9*, were significantly affected by GSO administration (Figure 8). Treatment with either crude grape seed oil (GSO) or its nanoemulsion (GSONE) restored the expression of these markers, with *Bax*, *TP53*, and *caspase-3* levels significantly higher in the EST/GSONE group compared to EST/GSO. At the same time, *caspase-9* did not show a statistically significant difference between the two treated groups. Conversely, the anti-apoptotic gene *Bcl-2* was markedly suppressed by both treatments, with no significant difference between the EST/GSO and EST/GSONE-treated groups.

**Figure 9:**
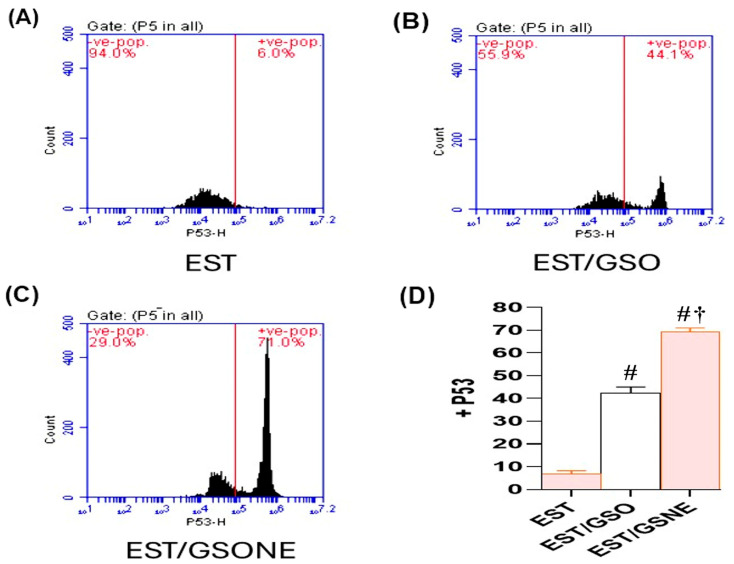
Flow cytometric analysis of p53 expression in Ehrlich solid tumor (EST) cells following treatment with grape seed oil formulations. (**A**–**C**) Representative dot plots showing p53-positive cell populations in untreated EST (**A**), EST treated with crude grape seed oil (GSO; (**B**)), and EST treated with grape seed oil nanoemulsion (GSONE; **C**). (**D**) Quantitative assessment demonstrates a significant, formulation-dependent increase in p53 expression, with GSONE treatment showing the greatest induction (*p* < 0.05 vs. EST and EST/GSO) (each group *n* = 10). Data are presented as mean ± SD. # Significantly different from the EST group; † Significantly different from the EST/GSO group.

Flow cytometric analysis (Figure 9A–C) further confirmed these findings at the cellular level: untreated ESTs demonstrated only 6.0% p53-positive cells, indicating minimal apoptosis. Treatment with GSO increased p53 positivity to 44.1%, while GSONE elevated this fraction to 71.0%, demonstrating significantly greater apoptotic activation than in both the EST and EST/GSO groups. Quantitative assessment (Figure 9D) showed that GSONE therapy was most effective at augmenting p53 expression and apoptotic cell frequency in tumor tissues.

**Figure 10:**
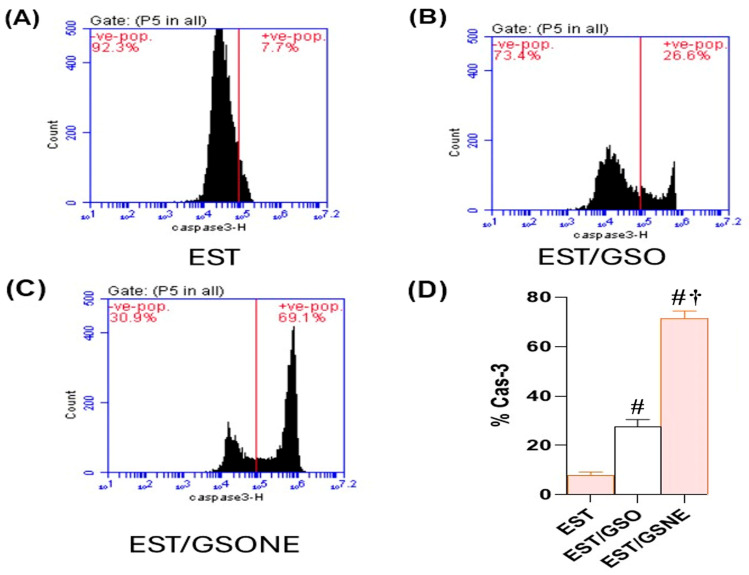
Flow cytometric assessment of cleaved caspase-3 expression in Ehrlich solid carcinoma (EST) cells following treatment with grape seed oil formulations. (**A**–**C**) Representative dot plots showing cleaved caspase-3-positive cell populations in untreated EST (**A**), EST treated with crude grape seed oil (GSO; (**B**)), and EST treated with grape seed oil nanoemulsion (GSONE; (**C**)). (**D**) Quantitative analysis demonstrates a significant, formulation-dependent increase in caspase-3 activation, with GSONE treatment yielding the highest apoptotic index (*p* < 0.05 vs. EST and EST/GSO) (each group *n* = 10). Data are presented as mean ± SD. # Significantly different from the EST group; † Significantly different from the EST/GSO group.

Flow cytometric assessment of cleaved caspase-3 in EST cells revealed a marked enhancement of apoptotic execution following treatment with grape seed oil formulations (Figure 10A–D). In the untreated EST group, only 7.7% of cells were positive for cleaved caspase-3, indicating minimal basal apoptotic activity. Administration of crude grape seed oil (EST/GSO) increased the proportion of cleaved caspase-3-positive cells to 26.6% (*p* < 0.05 vs. EST), suggesting moderate induction of apoptosis. Treatment with grape seed oil nanoemulsion (EST/GSONE) significantly elevated cleaved caspase-3 expression to 69.1% (*p* < 0.05 vs. both EST and EST/GSO), reflecting robust activation of the caspase-dependent apoptotic pathway. Quantitative analysis (Figure 10D) confirmed that GSONE conferred the highest apoptotic index, followed by GSO and the untreated control, highlighting the superior pro-apoptotic efficacy of nanoemulsion formulation.

### 3.8. Liver Histopathology and Ultrastructural Changes

**Figure 11:**
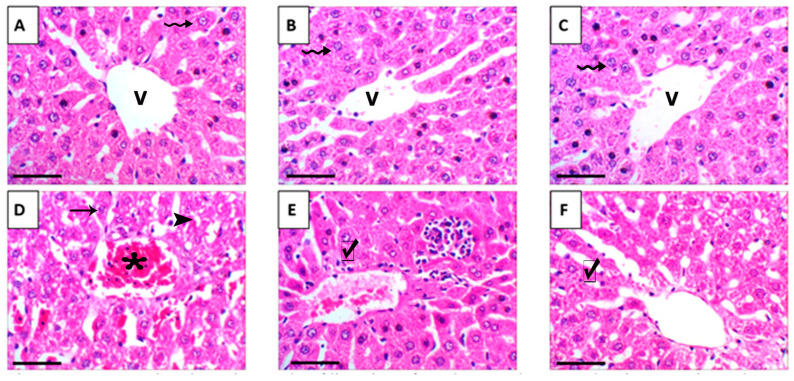
Representative photomicrographs of liver tissue from the control group and various experimental groups. (**A**) Control group: exhibits normal hepatic architecture that has a central vein (V) and healthy hepatocytes (wavy arrow). (**B**) GSO: grape seed oil (4 mg/kg body weight); shows normal hepatic architecture with central vein (V) and healthy hepatocytes (wavy arrow). (**C**) GSONE: grape seed oil nanoemulsion (4 mg/kg body weight); displays normal hepatic architecture with central vein (V) and healthy hepatocytes (wavy arrow). (**D**) EST: inoculated with Ehrlich ascites carcinoma (EAC) cells (0.2 mL); demonstrates vacuolar degeneration (arrowheads), central vein congestion (asterisk), and severe nuclear necrosis (arrow). (**E**) EST/GSO: grape seed oil (4 mg/kg body weight) + EAC cells (0.2 mL); shows restored liver architecture with well-formed hepatic cords (checkmark). (**F**) EST/GSONE: grape seed oil nanoemulsion (4 mg/kg body weight) + EAC cells (0.2 mL) shows well-formed hepatic cords (checkmark). All images were captured at 400× magnification; scale bar = 50 µm.

**Figure 12:**
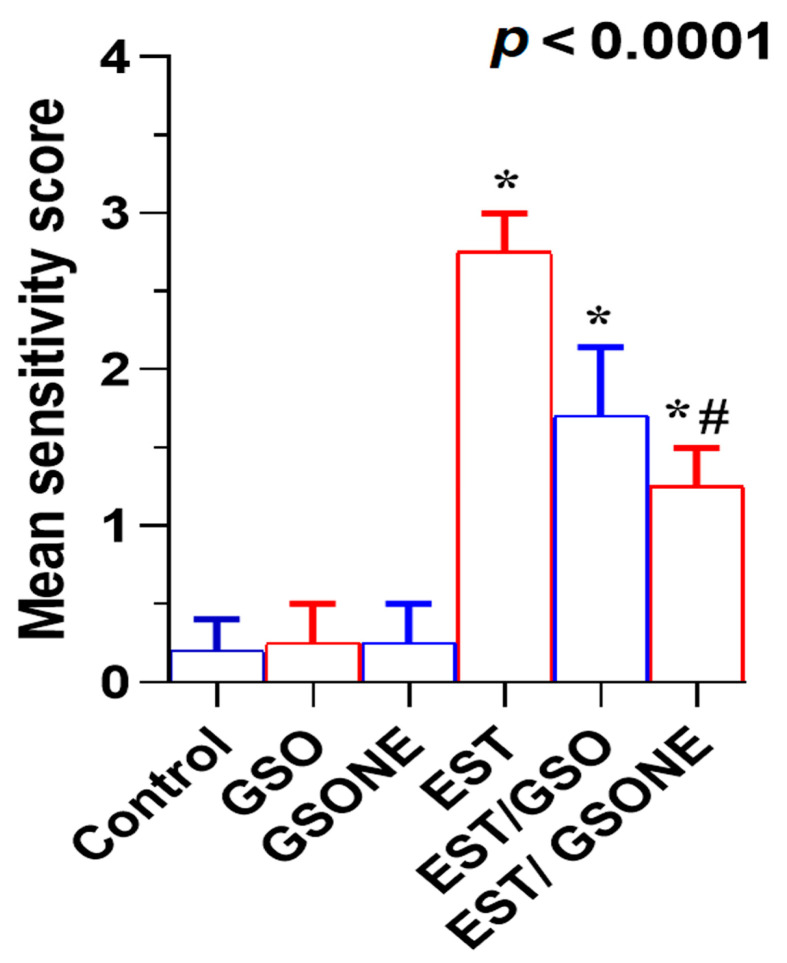
Mean severity scores of liver tissue damage in Ehrlich solid tumor (EST)-bearing mice treated with crude grape seed oil (GSO) or grape seed oil nanoemulsion (GSONE). Liver injury was assessed by semi-quantitative histopathological scoring in control, EST, EST/GSO, and EST/GSONE groups. GSO: Grape seed oil (4 mg/kg body weight); GSONE: Grape seed oil nanoemulsion (4 mg/kg body weight); EST: Inoculated with Ehrlich solid tumor cells (0.2 mL); EST/GSO: GSO + EST cells; EST/GSONE: GSONE + EST cells. Data are presented as mean ± SE (each group *n* = 10). Statistical differences between groups were determined by one-way ANOVA and Tukey’s post hoc test. * Significantly different from the control group; # Significantly different from the EST group.

Histological evaluation of liver tissue showed that mice treated with either crude grape seed oil (GSO) or its nanoemulsion form (GSONE) exhibited normal hepatic architecture, including intact central veins, well-organized lobular structures, and healthy hepatocytes with centrally positioned nuclei and uniform cytoplasmic morphology (Figure 11A–C). In contrast, hepatic tissue from mice bearing Ehrlich solid tumors (EST) demonstrated pronounced pathological changes, vacuolar degeneration, central vein congestion, and severe nuclear necrosis (Figure 11D). Treatment with GSO or GSONE in EST-bearing mice restored liver architecture, evidenced by well-formed hepatic cords and lobular structures (Figure 11E,F), indicating notable protection against tumor-induced damage. Quantitative scoring of liver injury confirmed marked injury in the EST group compared with controls. At the same time, GSONE treatment was particularly effective at alleviating tissue injury severity, with no significant difference in histopathological scores between the EST/GSO and EST/GSONE groups (Figure 12).

**Figure 13:**
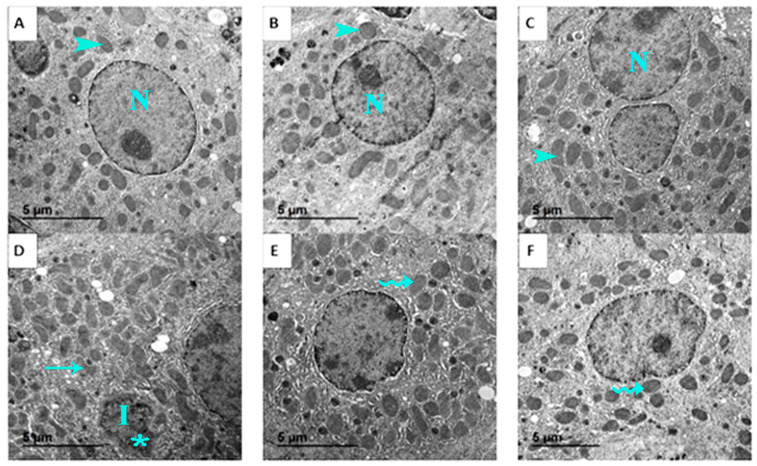
Representative transmission electron micrographs showing the liver ultrastructure from the control and experimental groups. (**A**) Control group: shows normal hepatocyte ultrastructure with nucleus (N) and healthy mitochondria (arrowhead). (**B**) GSO: grape seed oil (4 mg/kg body weight); maintains normal hepatocyte ultrastructure with nucleus (N) and healthy mitochondria (arrowhead). (**C**) GSONE: grape seed oil nanoemulsion (4 mg/kg body weight); maintains normal hepatocyte ultrastructure with nucleus (N) and healthy mitochondria (arrowhead). (**D**) EST: inoculated with Ehrlich ascites carcinoma (EAC) cells (0.2 mL); exhibits classic signs of cellular necrosis, including irregular nuclei (I), chromatin condensation (asterisk), extensive vacuolation, cytoplasmic degeneration, and disrupted mitochondrial cristae (arrow). (**E**) EST/GSO: grape seed oil (4 mg/kg body weight) + EAC cells (0.2 mL); shows restoration of near-normal ultrastructure with limited mitochondrial degeneration (wavey arrow). (**F**) EST/GSONE: grape seed oil nanoemulsion (4 mg/kg body weight) + EAC cells (0.2 mL) shows limited mitochondrial degeneration (wavy arrow), highlighting an observable hepatoprotective effect.

Ultrastructural analysis further supported these findings. Normal hepatocyte ultrastructure, including organized mitochondria, rough endoplasmic reticulum, and nuclei with distinct nucleoli, was maintained in control, GSO, and GSONE groups (Figure 13A–C). EST-bearing mice showed classic signs of cellular necrosis, irregular nuclei, chromatin condensation, extensive vacuolation, cytoplasmic degeneration, and disrupted mitochondrial cristae (Figure 13D). Restoration of near-normal ultrastructure, characterized by limited mitochondrial degeneration and preservation of organelle integrity, was observed in mice treated with either GSO or GSONE after EST induction (Figure 13E,F), highlighting the treatments’ hepatoprotective effects.

### 3.9. Multivariable Analyses

**Figure 14:**
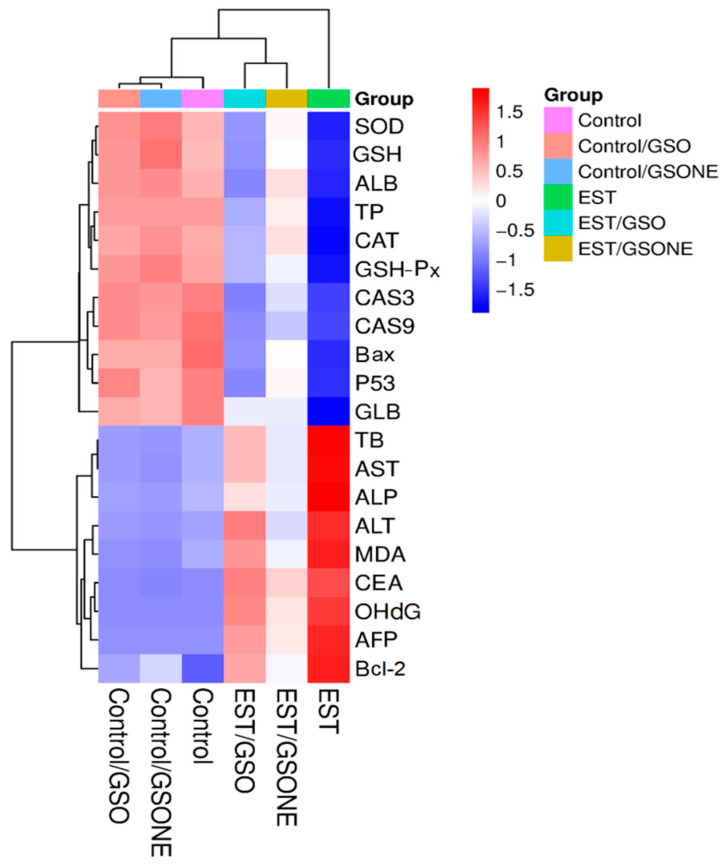
Hierarchical clustering heatmap of biochemical and molecular markers across experimental groups. Color gradients from red (upregulation) to blue (downregulation) reflect the relative abundance of markers, while clustering highlights the relationships among control, treatment-only, tumor-bearing, and combined treatment/tumor groups. GSO: Grape seed oil (4 mg/kg body weight); GSONE: Grape seed oil nanoemulsion (4 mg/kg body weight); EST: Inoculated with Ehrlich Ascites Carcinoma (EAC) cells (0.2 mL); EST/GSO: GSO + EST cells; EST/GSONE: GSONE + EST cells. Each group (*n* = 10). SOD: Superoxide dismutase; GSH: Reduced glutathione; AlB: Albumin; TP: Total protein; CAT: Catalase; GSH-Px: Glutathione peroxidase; CAS3/9: Caspase 3/9; Bax: Bcl-2-associated × protein; P53: Oncoprotein 53; GlB: Globulin; TB: Total bilirubin; AST: Aspartate transaminase; ALP: Alkaline phosphatase; ALT: Alanine transaminase; MDA: malondialdehyde; CEA: Cancer embroynic antigen; OhdG: Hydroxy-2′-deoxyguanosine; AFP: alpha feto protein; Bcl-2: B-cell lymphoma 2.

Hierarchical clustering heatmap analysis (Figure 14) delineated distinct biochemical and molecular expression profiles across experimental groups. The control and non-tumor treatment groups (Control/GSO, Control/GSONE) clustered together, characterized by elevated antioxidant enzyme activities (SOD, CAT, GSHP-x, GSH), albumin (ALB), and total protein (TP), alongside lower levels of pro-apoptotic and oxidative stress markers. In contrast, the EST group formed a separate cluster, characterized by diminished antioxidant parameters and elevated liver enzymes (AST, ALT, ALP), oxidative stress indices, and apoptotic markers, reflecting the metabolic and cellular disruption induced by tumor burden. Groups treated post-tumor induction (EST/GSO, EST/GSONE) occupied intermediate positions in the heatmap, demonstrating partial restoration of antioxidant status and suppression of tumor-related markers. Notably, the EST/GSONE group exhibited a more pronounced shift toward the healthy control profile than the EST/GSO group, highlighting the superior modulatory and therapeutic efficacy of the grape seed oil nanoemulsion formulation.

**Figure 15:**
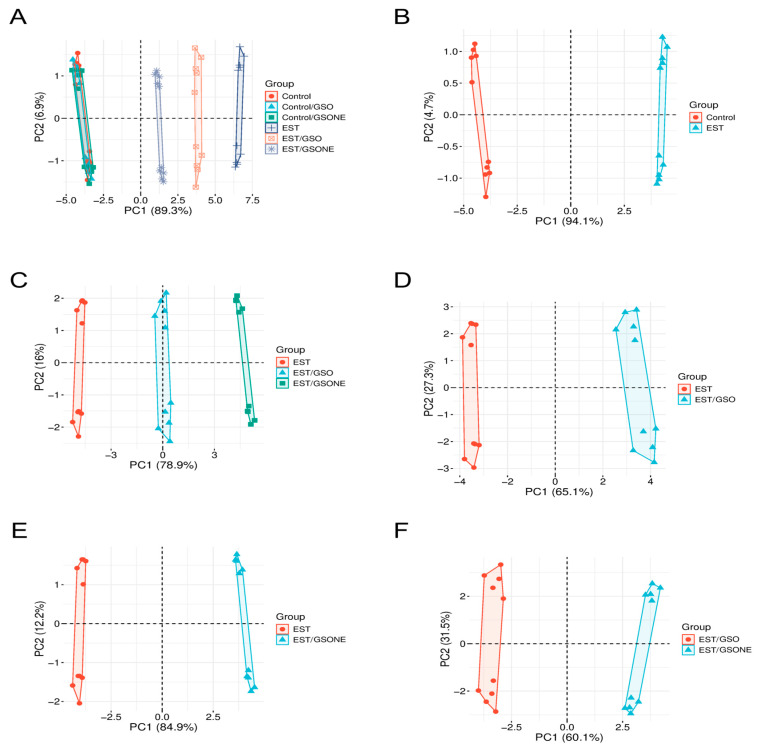
Principal component analysis (PCA) of biochemical and molecular profiles across experimental groups. (**A**) PCA plot showing the distribution of all experimental groups: Control, Control/GSO, Control/GSONE, EST, EST/GSO, and EST/GSONE; (**B**) PCA plot comparing Control and EST groups; (**C**) PCA plot comparing EST, EST/GSO, and EST/GSONE groups; (**D**) PCA plot comparing EST and EST/GSO groups. (**E**) PCA plot comparing EST and EST/GSONE groups. (**F**) PCA plot comparing EST/GSO and EST/GSONE groups. PCA plots display the multivariate distribution of samples, with colored points representing individual animals and ellipses denoting group clustering. GSO: Grape seed oil (4 mg/kg body weight); GSONE: grape seed oil nanoemulsion (4 mg/kg body weight); EST: Ehrlich solid tumor; EST/GSO: GSO + EST cells; EST/GSONE: GSONE + EST cells.

Principal component analysis (Figure 15) was performed to visualize multivariate differentiation among experimental groups based on biochemical and molecular features. The first principal component (PC1) accounted for 89.3% of the total variance (Figure 15A), clearly separating the tumor-bearing EST group from control and treatment-only cohorts, with healthy animals treated with GSO or GSONE clustering closely with controls. Pairwise PCA comparisons reinforced these distinctions: Figure 15B demonstrates near-complete segregation between the control and EST groups along PC1 (94.1%), indicating substantial tumor-induced biochemical shifts. Comparison of EST and EST/GSO (Figure 15C) revealed distinct clusters. Collectively, these multivariate results demonstrate that both GSO and GSONE confer biochemical protection in tumor-bearing mice, with the nanoemulsion formulation exhibiting superior restorative capacity.

## 4. Discussion

Ehrlich carcinoma remains a robust and widely used experimental tumor model that effectively mimics several biological and histopathological features of aggressive human cancers, notably rapid proliferation, low differentiation, and high responsiveness to antitumor intervention [25]. The evolution of this tumor model is known to induce oxidative stress, a hallmark of many malignancies, where an imbalance between oxidant production and antioxidant defenses results in DNA damage, lipid peroxidation, and mitochondrial dysfunction [42]. Such a redox imbalance not only promotes mutagenesis but also contributes to liver injury and systemic metabolic disruption. Therefore, counteracting oxidative stress using dietary antioxidants and phytochemicals represents a promising strategy for both cancer prevention and supportive therapy [43]. Therefore, counteracting oxidative stress using dietary antioxidants and phytochemicals represents a promising strategy for both cancer prevention and supportive therapy [44].

Extensive evidence has established the chemopreventive and therapeutic effects of grape seed–derived compounds in different cancer models [45,46]. Studies in colon, prostate, lung, and skin cancer models have demonstrated that grape seed extracts (GSEs) rich in polyphenols and proanthocyanidins inhibit proliferation, induce apoptosis, and suppress angiogenesis through pathways involving PI3K/Akt, MAPK, and NF-κB signaling [47,48,49,50,51,52,53,54]. GSO shares similar bioactivity, attributed to its unique composition of linoleic acid, tocopherols, phytosterols, and polyphenols, which exhibit pro-apoptotic and antioxidant mechanisms [55]. However, despite its promising biological activity, GSO’s poor aqueous solubility and oxidative instability have limited its practical use in biomedical formulations [23].

The incorporation of GSO into a nanoemulsion (GSONE) provides clear advantages in improving its stability, dispersion, and bioavailability [56,57]. Nanocarrier-based systems, including oil-in-water nanoemulsions, have demonstrated enhanced anticancer efficacy by facilitating cellular uptake and controlled release of hydrophobic phytochemicals [58]. In the present study, the nanoformulation exhibited a mean particle size of ~88 nm and zeta potential of −28 mV, parameters reported to favor intestinal permeability and cellular internalization [59,60,61,62,63]. These physicochemical characteristics are consistent with earlier nanoemulsion studies that improved the antitumor activity of other natural oils by enhancing free radical scavenging and mitochondrial uptake [64,65,66,67].

This study’s findings that GSONE reduced tumor volume and weight more effectively than crude GSO are in agreement with reports demonstrating that nanoencapsulation potentiates the cytotoxic and apoptotic actions of grape polyphenols [68,69]. Zhu et al. and Al-Ashmawy et al. similarly observed that GSO and proanthocyanidin-rich extracts decreased tumor cell viability by modulating caspase-dependent apoptosis and VEGF-linked angiogenesis [46,70]. The improved therapeutic activity of GSONE may therefore result from enhanced intracellular delivery of active lipophilic constituents that upregulate pro-apoptotic signals (Bax, TP53, caspase-3, caspase-9) and downregulate anti-apoptotic markers (Bcl-2), thereby promoting the intrinsic apoptotic cascade. Comparable mitochondrial apoptotic activation has been described for GSE, where proanthocyanidins increased cytochrome c release and stimulated caspase-3 cleavage in cervical cancer and colon cancer cells [71,72].

Biochemical restoration observed in this study also supports the hepatoprotective potential of GSONE. Elevated AST, ALT, ALP, and bilirubin levels are hallmarks of hepatic stress in tumor-bearing mice. Normalization of these parameters after GSONE treatment mirrors earlier findings, demonstrating that GSO restores hepatic antioxidant capacity in hepatotoxic models [73]. Moreover, the observed reductions in tumor biomarkers AFP and CEA are consistent with previous studies showing that polyphenolic grape derivatives improve liver integrity and reduce systemic oxidative stress [36,74,75].

Reduction in oxidative stress biomarkers (MDA, 8-OHdG) and concomitant elevation of endogenous antioxidants (SOD, CAT, GPx, GSH) demonstrate the enhancement of antioxidant defense mechanisms by GSONE, reinforcing the activation of Nrf2/ARE signaling known from other grape-derived interventions [76,77]. Similar redox modulation has been observed in GSE-treated models of head/neck and colon cancer, where reduced ROS generation coincided with improved mitochondrial function and DNA repair [78,79]. These antioxidant responses not only mitigate tumor-associated metabolic disturbance but also limit damage to hepatic and systemic tissues [80].

The multivariate analysis further consolidated the superiority of the nanoemulsion system relative to crude oil, showing distinct clustering toward healthy control profiles. Such global biochemical normalization underscores the systemic benefits of improved delivery systems in functional nutrient-based chemoprevention [81,82]. Comparable behavior has been reported in studies utilizing lipid-based nanoparticles containing grape seed bioactives, which demonstrated selective cytotoxicity toward tumor cells while sparing normal cell lines [83,84,85].

Collectively, this work situates GSONE within the broader context of grape-derived phytochemicals and nanotechnology-based delivery systems for cancer management. Previous investigations have confirmed that grape seed components act through multiple converging mechanisms, including antioxidant, anti-inflammatory, pro-apoptotic, and antiangiogenic effects. The present findings add value by demonstrating that nanoformulation magnifies these effects in vivo, producing superior restoration of hepatic and systemic homeostasis.

Several limitations should be acknowledged. The EST model, while well-validated, represents a single aggressive tumor type that may not fully reflect the heterogeneity of human cancers. The present study focused on one specific nanoemulsion formulation; variations in particle size, surfactant composition, or preparation methods could yield different outcomes. The short observation period assessed acute effects but precluded evaluation of long-term safety and sustained therapeutic benefits. Additionally, a direct comparison with standard chemotherapeutic agents was not conducted, limiting the assessment of relative therapeutic potential. While enhanced bioavailability was demonstrated, the precise mechanisms underlying nanoemulsion-mediated tissue distribution and cellular uptake remain to be fully elucidated. Another important limitation is that the phytochemical composition of the grape seed oil (GSO) used in this study was not experimentally analyzed in our laboratory. The interpretation of its chemical profile and bioactive constituents was based on previously published data rather than direct analysis, which may introduce minor variations in reproducibility or compositional consistency. Also, the transcriptomic changes underlying the effects of grape seed oil and nanoemulsion were evaluated using targeted qPCR rather than RNA sequencing (RNA-seq). Although qPCR provided specific mechanistic insights into apoptosis and oxidative stress regulation, RNA-seq would offer a comprehensive, genome-wide view of the signaling pathways modulated by the treatments. Due to current budgetary and time constraints, this approach was not pursued, but it remains an essential future direction for validating and extending the present findings.

Similarly, flow cytometric analysis of apoptosis markers (p53, cleaved caspase-3) was performed on dispersed tumor cells without additional gating for epithelial markers such as EpCAM or cytokeratin, thereby restricting analysis to tumor cell populations. Moreover, while the observed loss of viable cells reflected the cytotoxic effects of GSO and GSONE, immunohistochemistry (IHC) or immunofluorescence (IF) could provide improved spatial localization and quantitative confirmation of apoptotic marker expression in tumor tissues.

Future research should aim to integrate omics-based profiling, long-term safety assessments, and direct mapping of nanocarrier-tumor interactions to fully delineate the molecular pathways engaged by grape seed oil nanoemulsions. By bridging nutrition, nanotechnology, and oncology, such approaches will advance the translational potential of dietary phytochemical nanoformulations for cancer prevention and adjunctive therapy.

## 5. Conclusions

This study demonstrates that GSONE confers greater protection against systemic oxidative stress and secondary hepatic injury induced by EST than crude GSO. The superior efficacy of GSONE can be attributed to its enhanced physicochemical properties, including improved stability, water solubility, and bioavailability, afforded by its nanoformulation. Mechanistically, GSONE acts through multiple complementary pathways, exerting potent antioxidant effects, restoring cellular redox balance, modulating oxidative stress-related biochemical parameters, and preserving liver histoarchitecture during tumor challenge. Importantly, GSONE also exhibits pronounced anti-apoptotic activity in tumor cells by downregulating some pro-apoptotic players and mitigating apoptosis-associated molecular disruptions, thereby highlighting its therapeutic promise for the management of malignancies driven by oxidative and metabolic stress.

These findings underscore the potential of nanoemulsion-based delivery systems to enhance the clinical utility of natural bioactives, such as grape seed oil, and support further translational investigation of GSONE as a novel adjunct in cancer therapy.

## Figures and Tables

**Table 1:** Sequences of Primers Employed in qRT-PCR for Target Gene Amplification.

Gene	Sequences (5′-3′)	Length (bp)
*Bax*	F: GTCTCCGGCGAATTGGAGAT R: ACCCGGAAGAAGACCTCTCG	100
*Bcl-2*	F: CATCGCCCTGTGGATGACTG R: GGCCATATAGTTCCACAAAGGC	95
*TP53*	F: CCCCTGTCATCTTTTGTCCCT R: AGCTGGCAGAATAGCTTATTGAG	137
*Caspase*-*3*	F: ACTGGAATGTCAGCTCGCAA R: GCAGTAGTCGCCTCTGAAGA	270
*Caspase*-*9*	F: ACGTGAACTTCTGCCCTTCC R: GGTCGTTCTTCACCTCCACC	117
*GAPDH*	F: GTATCGGACGCCTGGTTAC R: CTTGCCGTGGGTAGAGTCAT	128

F: Forward; R: Reverse; bp: base pair; *Bax*: Bcl-2-associated X protein; *Bcl-2*: B-cell lymphoma 2; *TP53*: Tumor Protein 53; *Caspase-3/9*: Cysteine-aspartic acid protease-3/9; *GAPDH*: Glyceraldehyde-3-phosphate dehydrogenase.

**Table 2:** Semi-quantitative scoring of hepatic damage was based on a four-point grading scale.

Score	Hepatic Lesions Were Graded Using an Integrated Semi-Quantitative Scoring System.
0 (none)	Histological analysis revealed normal architecture.
1 (mild)	Hepatocellular degeneration and necrosis were occasionally observed, accompanied by minimal or absent inflammatory infiltration and rare vascular congestion.
2 (moderate)	Moderate vacuolar degeneration, scattered inflammatory cell infiltration, multifocal hepatocellular necrosis, and mild blood vessel congestion were shown in the hepatic tissue.
3 (severe)	Diffuse and severe degenerative alterations were shown in hepatic tissue, along with widespread infiltration of leukocytes, extensive hepatocellular necrosis, and moderate to severe congestion of the hepatic vasculature.

**Table 3:** Growth performance and tumor weight changes induced by grape seed oil nanoemulsion compared to raw grape seed oil in mice bearing Ehrlich solid carcinoma.

Parameters	Control	GSO	GSONE	EST	EST/GSO	EST/GSONE	*p*-Values
IBW (g)	21.86 ± 0.33	21.69 ± 0.29	21.44 ± 0.26	21.93 ± 0.38	21.56 ± 0.37	21.38 ± 0.48	0.746
FBW (g)	27.46 ± 0.24	27.86 ± 0.31	27.93 ± 0.22	27.38 ± 0.19	26.87 ± 0.25	27.34 ± 0.18	0.041
NW(g)	27.46 ± 0.24	27.86 ± 0.31	27.93 ± 0.22	20.62 ± 0.19 *	22.43 ± 0.25 *#	24.16 ± 0.12 *#†	0.003
BWG (g)	5.60 ± 0.09	6.17 ± 0.02	6.49 ± 0.04	−1.30 ± 0.19 *	0.87 ± 0.12 *#	2.79 ± 0.37 *#†	<0.0001

GSO: Grape seed oil (4 mg/kg body weight); GSONE: Grape seed oil nanoemulsion (4 mg/kg body weight); EST: Inoculated with Ehrlich Ascites Carcinoma (EAC) cells (0.2 mL); EST/GSO: Grape seed oil (4 mg/kg body weight) + EAC cells (0.2 mL); EST/GSONE: Grape seed oil nanoemulsion (4 mg/kg body weight) + EAC cells (0.2 mL) (each group *n* = 15). IBW: Initial body weight; FBW: Final body weight; NW: Net Weight (g); body weight after subtraction of tumor mass in tumor-bearing groups; BWG: Body Weight Gain (g); calculated as NW—IBW to reflect net gain or loss excluding tumor weight; TM: Tumor. Values are presented as mean ± SE. * Significantly different from the control group; # Significantly different from the EST group; † Significantly different from the EST/GSO group. The significance level is *p* < 0.05.

**Table 4:** Blood biochemical changes induced by grape seed oil nanoemulsion compared to raw grape seed oil in mice bearing Ehrlich solid carcinoma.

Parameters	Control	GSO	GSONE	EST	EST/GSO	EST/GSONE	*p*-Values
TP (g/dL)	6.88 ± 0.29	6.90 ± 0.31	6.92 ± 0.19	4.13 ± 0.13 *	5.38 ± 0.32 *#	6.19 ± 0.27 #†	0.0146
Alb (g/dL)	3.79 ± 0.13	3.93 ± 0.17	3.98 ± 0.21	2.16 ± 0.07 *	2.71 ± 0.12 *#	3.53 ± 0.18 #†	0.0273
Glo (g/dL)	3.09 ± 0.16	2.97 ± 0.14	2.94 ± 0.06	1.97 ± 0.06 *	2.67 ± 0.10 #	2.66 ± 0.09 #	0.0047
TB (mg/dL)	0.42 ± 0.09	0.38 ± 0.07	0.36 ± 0.06	1.32 ± 0.25 *	0.86 ± 0.03 *#	0.58 ± 0.10 #	<0.0001
ALP (U/L)	88.32 ± 4.26	86.25 ± 5.18	85.41 ± 6.22	128.14 ± 7.52 *	101.21 ± 6.91 #	95.33 ± 4.93 #	0.0026
ALT (U/L)	46.26 ± 3.24	45.21 ± 4.35	45.03 ± 5.11	89.25 ± 4.89 *	78.29 ± 6.23 *	54.26 ± 5.39 #†	0.0001
AST (U/L)	76.32 ± 6.66	73.41 ± 4.20	72.27 ± 5.38	118.39 ± 6.15 *	96.20 ± 4.44 *#	84.06 ± 5.93 #	0.0001

GSO: Grape seed oil (4 mg/kg body weight); GSONE: Grape seed oil nanoemulsion (4 mg/kg body weight); EST: Inoculated with Ehrlich Ascites Carcinoma (EAC) cells (0.2 mL); EST/GSO: Grape seed oil (4 mg/kg body weight) + EAC cells (0.2 mL); EST/GSONE: Grape seed oil nanoemulsion (4 mg/kg body weight) + EAC cells (0.2 mL). Values are presented as mean ± SE, with each group (*n* = 10). TP: Total protein; Alb: Albumin; Glo: Globulin; TB: Total Bilirubin; ALP: Alkaline phosphatase; ALT: Alanine transaminase; AST: Aspartate transaminase. * Significantly different from the control group; # Significantly different from the EST group; † Significantly different from the EST/GSO group. The significance level is *p* < 0.05.

## Data Availability

The original contributions presented in this study are included in the article. Further inquiries can be directed to the corresponding author.

## Abbreviations

The following abbreviations are used in this manuscript:

ALT	Alanine aminotransferase
ALP	Alkaline phosphatase
AFP	Alpha fetoprotein
AST	Aspartate aminotransferase
Bax	Bcl-2-associated x protein
Bcl-2	B-cell lymphoma 2
CEA	Carcinoembryonic antigen
CAT	Catalase
cDNA	Complementary DNA
DLS	Dynamic light scattering
EAC	Ehrlich Ascites Carcinoma
ELFA	Enzyme-linked fluorescent assay
EST	Ehrlich solid Tumor
GAPDH	Glyceraldehyde phosphate dehydrogenase
GSH	Glutathione (reduced)
GSO	Grape seed oil
GSONE	Grape seed oil nanoemulsion
GSH-Px	Glutathione peroxidase
H&E	Hematoxylin and eosin
MDA	Malondialdehyde
NCI	National Cancer Institute
p53	Oncoprotein 53
PBS	Phosphate-buffered saline
PCA	Principal component analysis
PDI	Polydispersity index
PE	Phycoerythrin
SE	Standard error
SOD	Superoxide dismutase
TBARS	Thiobarbituric acid reactive substances
TEM	Transmission electron microscopy
TP53	Tumor protein 53 gene
TP	Total protein

## References

[1] Atuahene D., Mahama K., Sam B.A., Appiah D.A., Pandey V.K., Bela K., Harsányi E., Shaikh A.M. (2025). Dietary targeting of cancer pathways: Role of bioactive compounds and nutraceuticals. Food Humanit..

[2] Li L., Jin P., Guan Y., Luo M., Wang Y., He B., Li B., He K., Cao J., Huang C. (2022). Exploiting Polyphenol-Mediated Redox Reorientation in Cancer Therapy. Pharmaceuticals.

[3] Rudzińska A., Juchaniuk P., Oberda J., Wiśniewska J., Wojdan W., Szklener K., Mańdziuk S. (2023). Phytochemicals in Cancer Treatment and Cancer Prevention-Review on Epidemiological Data and Clinical Trials. Nutrients.

[4] Rezagholizade-shirvan A., Soltani M., Shokri S., Radfar R., Arab M., Shamloo E. (2024). Bioactive compound encapsulation: Characteristics, applications in food systems, and implications for human health. Food Chem. X.

[5] Clemente-Suárez V.J., Bustamante-Sanchez A., Rubio-Zarapuz A., Martín-Rodríguez A., Tornero-Aguilera J.F., Beltrán-Velasco A.I. (2025). Biomimetic Strategies for Nutraceutical Delivery: Advances in Bionanomedicine for Enhanced Nutritional Health. Biomimetics.

[6] Poureshaghi F., Eghlima G., Khanmohammadi D., Esmaeili H., Mirjalili M.H. (2025). Variability in seed oil content, fatty acids profile, phytochemical properties, mineral and proximate composition of Iranian cultivars of *Vitis vinifera* L.. Sci. Rep..

[7] Lin Z., Grasso S. (2025). Exploring seed-based upcycled oils: Types, extraction processes, and emerging applications. Crit. Rev. Food Sci. Nutr..

[8] de Almeida Sousa Cruz M.A., de Barros Elias M., Calina D., Sharifi-Rad J., Teodoro A.J. (2024). Insights into grape-derived health benefits: A comprehensive overview. Food Prod. Process. Nutr..

[9] Dabetic N., Todorovic V., Djuricic I., Stankovic J., Basić Z., Vujovic D., Sobajic S. (2020). Grape Seed Oil Characterization: A Novel Approach for Oil Quality Assessment. Eur. J. Lipid Sci. Technol..

[10] Zhao L., Yagiz Y., Xu C., Fang X., Marshall M.R. (2017). Identification and characterization of vitamin E isomers, phenolic compounds, fatty acid composition, and antioxidant activity in seed oils from different muscadine grape cultivars. J. Food Biochem..

[11] Bellili S., Jazi S., Nasr S., Dhifi W., Neves M.A., Miguel M.G.C., Mnif W. (2018). Grape Seed Oil: Chemical Composition, Biological Properties and Health Benefits.

[12] Mahanna M., Millan-Linares M.C., Grao-Cruces E., Claro C., Toscano R., Rodriguez-Martin N.M., Naranjo M.C., Montserrat-de la Paz S. (2019). Resveratrol-enriched grape seed oil (*Vitis vinifera* L.) protects from white fat dysfunction in obese mice. J. Funct. Foods.

[13] Zhang L., Chen J., Liang R., Liu W., Chen M., Chen J. (2022). Synergistic Anti-Inflammatory Effects of Lipophilic Grape Seed Proanthocyanidin and Camellia Oil Combination in LPS-Stimulated RAW264.7 Cells. Antioxidants.

[14] Sánchez-López E., Guerra M., Dias-Ferreira J., Lopez-Machado A., Ettcheto M., Cano A., Espina M., Camins A., Garcia M.L., Souto E.B. (2019). Current Applications of Nanoemulsions in Cancer Therapeutics. Nanomaterials.

[15] Ahmed S.A.A., Mahsoub F., El Gamal S.A., Khamis T., Faroh K.Y., Abdelwarith A.A., Younis E.M., Saad M.F., Ali H.S., Davies S.J. (2025). Chitosan-grape seed oil nanoemulsion enriched diet promotes performance, antioxidant-immune metrics and modifies immune- gene action and morphological architecture in Nile tilapia against Aeromonas veronii. Aquac. Rep..

[16] Gupta M., Dey S., Marbaniang D., Pal P., Ray S., Mazumder B. (2020). Grape seed extract: Having a potential health benefits. J. Food Sci. Technol..

[17] Castro M.L., Azevedo-Silva J., Valente D., Machado A., Ribeiro T., Ferreira J.P., Pintado M., Ramos O.L., Borges S., Baptista-Silva S. (2024). Elevating Skincare Science: Grape Seed Extract Encapsulation for Dermatological Care. Molecules.

[18] Rached R.A., Habre M., Salem Y., Khodeir J., Allaw M., Castangia I., Rajha H.N., Habre L., Feghali J., Touma J.A. (2025). Clinical Trial to Evaluate the Effect of Grape Seed Extract-Loaded Hyalurosomes on Skin Wellness. Cosmetics.

[19] Nateghi L., Hosseini E. (2023). Investigating the oxidative stability of grape seed oil using aqueous extract of pistachio green hull. J. Food Meas. Charact..

[20] Bhutani M., Gaur S.S., Shams R., Dash K.K., Shaikh A.M., Béla K. (2025). Valorization of grape by-products: Insights into sustainable industrial and nutraceutical applications. Future Foods.

[21] Böger B., Georgetti S., Kurozawa L. (2018). Microencapsulation of grape seed oil by spray drying. Food Sci. Technol..

[22] Mundo J.L.M., Zhou H., Tan Y., Liu J., McClements D.J. (2021). Enhancing emulsion functionality using multilayer technology: Coating lipid droplets with saponin-polypeptide-polysaccharide layers by electrostatic deposition. Food Res. Int..

[23] Sepeidnameh M., Fazlara A., Hosseini S.M.H., Pourmahdi Borujeni M. (2024). Encapsulation of grape seed oil in oil-in-water emulsion using multilayer technology: Investigation of physical stability, physicochemical and oxidative properties of emulsions under the influence of the number of layers. Curr. Res. Food Sci..

[24] Mutlu N. (2023). Effects of grape seed oil nanoemulsion on physicochemical and antibacterial properties of gelatin-sodium alginate film blends. Int. J. Biol. Macromol..

[25] Radulski D.R., Stipp M.C., Galindo C.M., Acco A. (2023). Features and applications of Ehrlich tumor model in cancer studies: A literature review. Transl. Breast Cancer Res..

[26] El-Masry T.A., El-Nagar M.M.F., El Mahdy N.A., Alherz F.A., Taher R., Osman E.Y. (2024). Potential Antitumor Activity of Combined Lycopene and Sorafenib against Solid Ehrlich Carcinoma via Targeting Autophagy and Apoptosis and Suppressing Proliferation. Pharmaceuticals.

[27] Dahran N., Othman M.S., Ghoniem M.E., Samak M.A., Elabbasy M.T., Obeidat S.T., Aleid G.M., Abo Elnaga S., Khaled A.M., Altaleb A.A. (2024). Evaluation of Vincamine Loaded with Silver Nanoparticles as a New Potential Therapeutic Agent Against Ehrlich’s Solid Carcinoma in Mice. Cells.

[28] Alfawaz M., Elmorsy E.M., Samy A., Shams A.S., Salem M.A., Shaalan A.A.M., Fawzy M.S., Hosny N. (2025). Therapeutic Potential of Food-Derived Rutin Phytosome Nanoparticles: Antitumor, Antioxidant, and Anti-Inflammatory Activity in Ehrlich Ascites Carcinoma. Pharmaceuticals.

[29] Li K., Deng Z., Lei C., Ding X., Li J., Wang C. (2024). The Role of Oxidative Stress in Tumorigenesis and Progression. Cells.

[30] Aldubayan M.A., Elgharabawy R.M., Ahmed A.S., Tousson E. (2019). Antineoplastic Activity and Curative Role of Avenanthramides against the Growth of Ehrlich Solid Tumors in Mice. Oxid. Med. Cell Longev..

[31] Sayed H.M., Said M.M., Morcos N.Y.S., El Gawish M.A., Ismail A.F.M. (2021). Antitumor and Radiosensitizing Effects of Zinc Oxide-Caffeic Acid Nanoparticles against Solid Ehrlich Carcinoma in Female Mice. Integr. Cancer Ther..

[32] Uti D.E., Atangwho I.J., Alum E.U., Ntaobeten E., Obeten U.N., Bawa I., Agada S.A., Ukam C.I., Egbung G.E. (2025). Antioxidants in cancer therapy mitigating lipid peroxidation without compromising treatment through nanotechnology. Discov. Nano.

[33] Yılmaz S., Doğanyiğit Z., Oflamaz A.O., Ateş Ş., Söylemez E.S.A., Nisari M., Farooqı A.A. (2023). Determination of Rutin’s antitumoral effect on EAC solid tumor by AgNOR count and PI3K/AKT/mTOR signaling pathway. Med. Oncol..

[34] Alotaibi B., Tousson E., El-Masry T.A., Altwaijry N., Saleh A. (2021). Ehrlich ascites carcinoma as model for studying the cardiac protective effects of curcumin nanoparticles against cardiac damage in female mice. Environ. Toxicol..

[35] Louis K.S., Siegel A.C. (2011). Cell viability analysis using trypan blue: Manual and automated methods. Methods Mol. Biol..

[36] Ismail A.F.M., Salem A.A.M., Eassawy M.M.T. (2016). Hepatoprotective effect of grape seed oil against carbon tetrachloride induced oxidative stress in liver of γ-irradiated rat. J. Photochem. Photobiol. B Biol..

[37] Sannappa Gowda N.G., Shiragannavar V.D., Prabhuswamimath S.C., Tuladhar S., Chidambaram S.B., Santhekadur P.K. (2022). Ehrlich Ascites carcinoma mice model for studying liver inflammation and fibrosis. Adv. Cancer Biol.-Metastasis.

[38] Eltahir Z., Ibrahim M., Mohieldeen M.Y., Bayoumi A., Ahmed S.M. (2024). Thymoquinone Nanoparticles (TQ-NPs) in Kidney Toxicity Induced by Ehrlich Ascites Carcinoma (EAC): An In Vivo Study. Can. J. Kidney Health Dis..

[39] Baris M.M., Serinan E., Calisir M., Simsek K., Aktas S., Yilmaz O., Ozdemir S.K., Secil M. (2020). Xenograft Tumor Volume Measurement in Nude Mice: Estimation of 3D Ultrasound Volume Measurements Based on Manual Caliper Measurements. J. Basic Clin. Health Sci..

[40] Mohamed H.R.H., Tulbah F.S.A., El-ghor A.A., Eissa S.M. (2023). Suppression of tumor growth and apoptosis induction by pomegranate seed nanoemulsion in mice bearing solid Ehrlich carcinoma cells. Sci. Rep..

[41] Gencer S., Gür C., İleritürk M., Küçükler S., Akaras N., Şimşek H., Kandemir F.M. (2024). The ameliorative effect of carvacrol on sodium arsenite-induced hepatotoxicity in rats: Possible role of Nrf2/HO-1, RAGE/NLRP3, Bax/Bcl-2/Caspase-3, and Beclin-1 pathways. J. Biochem. Mol. Toxicol..

[42] Chandimali N., Bak S.G., Park E.H., Lim H.-J., Won Y.-S., Kim E.-K., Park S.-I., Lee S.J. (2025). Free radicals and their impact on health and antioxidant defenses: A review. Cell Death Discov..

[43] Chaudhary P., Janmeda P., Docea A.O., Yeskaliyeva B., Abdull Razis A.F., Modu B., Calina D., Sharifi-Rad J. (2023). Oxidative stress, free radicals and antioxidants: Potential crosstalk in the pathophysiology of human diseases. Front. Chem..

[44] Liang X., Weng J., You Z., Wang Y., Wen J., Xia Z., Huang S., Luo P., Cheng Q. (2025). Oxidative stress in cancer: From tumor and microenvironment remodeling to therapeutic frontiers. Mol. Cancer.

[45] Sochorova L., Prusova B., Cebova M., Jurikova T., Mlcek J., Adamkova A., Nedomova S., Baron M., Sochor J. (2020). Health Effects of Grape Seed and Skin Extracts and Their Influence on Biochemical Markers. Molecules.

[46] Al-Ashmawy G.M., Labah D.A., Wahba O.M., Abdel Ghafar M.T., El-Feky O.A. (2023). Cancer chemopreventive role of grape seed oil and cisplatin as a combination adjuvant therapy in the treatment of tongue squamous cell carcinoma: A biological in-vitro study. Arch. Oral Biol..

[47] Porcelli L., Iacobazzi R.M., Quatrale A.E., Bergamini C., Denora N., Crupi P., Antonacci D., Mangia A., Simone G., Silvestris N. (2017). Grape seed extracts modify the outcome of oxaliplatin in colon cancer cells by interfering with cellular mechanisms of drug cytotoxicity. Oncotarget.

[48] Hamza A.A., Heeba G.H., Elwy H.M., Murali C., El-Awady R., Amin A. (2018). Molecular characterization of the grape seeds extract’s effect against chemically induced liver cancer: In vivo and in vitro analyses. Sci. Rep..

[49] Chen M., Yu S. (2019). Lipophilic Grape Seed Proanthocyanidin Exerts Anti-Proliferative and Pro-Apoptotic Effects on PC3 Human Prostate Cancer Cells and Suppresses PC3 Xenograft Tumor Growth in Vivo. J. Agric. Food Chem..

[50] Suganya M., Gnanamangai B.M., Ravindran B., Chang S.W., Selvaraj A., Govindasamy C., Elsadek M.F., Ponmurugan P. (2019). Antitumor effect of proanthocyanidin induced apoptosis in human colorectal cancer (HT-29) cells and its molecular docking studies. BMC Chem..

[51] Wang L., Zhan J., Huang W. (2020). Grape Seed Proanthocyanidins Induce Apoptosis and Cell Cycle Arrest of HepG2 Cells Accompanied by Induction of the MAPK Pathway and NAG-1. Antioxidants.

[52] Gašić U., Ćirić I., Pejčić T., Radenković D., Djordjević V., Radulović S., Tešić Ž. (2020). Polyphenols as Possible Agents for Pancreatic Diseases. Antioxidants.

[53] Ahmed Z.S.O., Khan E., Elias N., Elshebiny A., Dou Q. (2025). Updated Review on Natural Polyphenols: Molecular Mechanisms, Biological Effects, and Clinical Applications for Cancer Management. Biomolecules.

[54] Hirsa M., Fichna J., Tarasiuk-Zawadzka A. (2025). Phytotherapy with Fruit Seed Extracts as a Promising Approach for the Treatment of Inflammation. Curr. Nutr. Rep..

[55] Cravotto C., Rapinel V., Nguyen-Thanh B., Bonet-García R., Bartier M., Claux O., Jacques L., Tabasso S., Barrajón-Catalán E., Fabiano-Tixier A.-S. (2025). Sustainable grape seed oil processing: Green solvent extraction and by-product valorisation. Food Bioprod. Process..

[56] Preeti, Sambhakar S., Malik R., Bhatia S., Al Harrasi A., Rani C., Saharan R., Kumar S., Geeta, Sehrawat R. (2023). Nanoemulsion: An Emerging Novel Technology for Improving the Bioavailability of Drugs. Scientifica.

[57] Tanuku S., Velisila D., Thatraju D., Vadaga A. (2024). Nanoemulsion Formulation Strategies for Enhanced Drug Delivery: Review Article. J. Pharma Insights Res..

[58] Chavda V.P., Nalla L.V., Balar P., Bezbaruah R., Apostolopoulos V., Singla R.K., Khadela A., Vora L., Uversky V.N. (2023). Advanced Phytochemical-Based Nanocarrier Systems for the Treatment of Breast Cancer. Cancers.

[59] Honary S., Zahir F. (2013). Effect of Zeta Potential on the Properties of Nano-Drug Delivery Systems—A Review (Part 2). Trop. J. Pharm. Res..

[60] Ejazi S.A., Louisthelmy R., Maisel K. (2023). Mechanisms of Nanoparticle Transport across Intestinal Tissue: An Oral Delivery Perspective. ACS Nano.

[61] Öztürk K., Kaplan M., Calis S. (2024). Effects of nanoparticle size, shape, and zeta potential on drug delivery. Int. J. Pharm..

[62] Cahyani D.M., Mubarok A.S., Hariawan B.S., Amalina I., Drake P., Parumasivam T., Sahu R.K., Rijal M.A.S., Sari R., Miatmoko A. (2025). Nanoparticle tools for maximizing oral drug delivery. Braz. J. Med. Biol. Res. Rev. Bras. Pesqui. Medicas E Biol..

[63] Guo S., Liang Y., Liu L., Yin M., Wang A., Sun K., Li Y., Shi Y. (2021). Research on the fate of polymeric nanoparticles in the process of the intestinal absorption based on model nanoparticles with various characteristics: Size, surface charge and pro-hydrophobics. J. Nanobiotechnol..

[64] Garavaglia J., Markoski M.M., Oliveira A., Marcadenti A. (2016). Grape Seed Oil Compounds: Biological and Chemical Actions for Health. Nutr. Metab. Insights.

[65] Yang B., Dong Y., Wang F., Zhang Y. (2020). Nanoformulations to Enhance the Bioavailability and Physiological Functions of Polyphenols. Molecules.

[66] Roozitalab G., Yousefpoor Y., Abdollahi A., Safari M., Rasti F., Osanloo M. (2022). Antioxidative, anticancer, and antibacterial activities of a nanoemulsion-based gel containing *Myrtus communis* L. essential oil. Chem. Pap..

[67] Seyhan V., Barla Demirkoz A., Ner M. (2023). Nanoemulsions: New Approaches in Cancer Therapy with Herbal Terpenes and Essential Oils.

[68] Rahaiee S., Assadpour E., Faridi Esfanjani A., Silva A.S., Jafari S.M. (2020). Application of nano/microencapsulated phenolic compounds against cancer. Adv. Colloid Interface Sci..

[69] Khatoon S., Kalam N., Shaikh M.F., Hasnain M., Hafiz A.K., Ansari M.T. (2021). Nanoencapsulation of Polyphenols as Drugs and Supplements for Enhancing Therapeutic Profile—A Review. Curr. Mol. Pharmacol..

[70] Zhu F., Du B., Li J. (2015). Recent advance on the antitumor and antioxidant activity of grape seed extracts. Int. J. Wine Res..

[71] Chen Q., Liu X.-F., Zheng P.-S. (2014). Grape Seed Proanthocyanidins (GSPs) Inhibit the Growth of Cervical Cancer by Inducing Apoptosis Mediated by the Mitochondrial Pathway. PLoS ONE.

[72] Zhang C., Chen W., Zhang X., Zheng Y., Yu F., Liu Y., Wang Y. (2017). Grape seed proanthocyanidins induce mitochondrial pathway-mediated apoptosis in human colorectal carcinoma cells. Oncol. Lett..

[73] Ali D.A., Badr El-Din N.K., Abou-El-magd R.F. (2015). Antioxidant and hepatoprotective activities of grape seeds and skin against Ehrlich solid tumor induced oxidative stress in mice. Egypt. J. Basic Appl. Sci..

[74] Madbouly N., Ali D., Farid A. (2025). Nanoparticles from grape seed extract inhibit inflammatory cytokines and ameliorate CCl4-induced hepatotoxicity. BMC Complement. Med. Ther..

[75] Alhajlah S. (2024). Effect of grape-derived products on the serum levels of enzymes mainly produced by the liver: A systematic review and meta-analysis of parallel randomized controlled trials. Phytother. Res. PTR.

[76] Ma X., Li Y., Lv C., Liu B., Yuan C., Huang W., Luo Q., Xiao Y., Sun C., Li T. (2022). Modulation of Keap1-Nrf2-ARE signaling pathway by oxyresveratrol, a derivative of resveratrol from grape skin. Food Biosci..

[77] Li H., Zhang H., Wang T., Zhang L., Wang H., Lu H., Yang R., Ding Y. (2024). Grape Seed Proanthocyanidins Protect Pancreatic β Cells Against Ferroptosis via the Nrf2 Pathway in Type 2 Diabetes. Biol. Trace Elem. Res..

[78] Shrotriya S., Deep G., Lopert P., Patel M., Agarwal R., Agarwal C. (2015). Grape seed extract targets mitochondrial electron transport chain complex III and induces oxidative and metabolic stress leading to cytoprotective autophagy and apoptotic death in human head and neck cancer cells. Mol. Carcinog..

[79] Pérez-Ortiz J.M., Alguacil L.F., Salas E., Hermosín-Gutiérrez I., Gómez-Alonso S., González-Martín C. (2019). Antiproliferative and cytotoxic effects of grape pomace and grape seed extracts on colorectal cancer cell lines. Food Sci. Nutr..

[80] Abd Eldaim M.A., Tousson E., Soliman M.M., El Sayed I.E.T., Abdel Aleem A.A.H., Elsharkawy H.N. (2021). Grape seed extract ameliorated Ehrlich solid tumor-induced hepatic tissue and DNA damage with reduction of PCNA and P53 protein expression in mice. Environ. Sci. Pollut. Res. Int..

[81] Yousefpoor Y., Esnaashari S.S., Baharifar H., Mehrabi M., Amani A. (2023). Current challenges ahead in preparation, characterization, and pharmaceutical applications of nanoemulsions. WIREs Nanomed. Nanobiotechnol..

[82] Goswami A., Rawat R., Pillai P., Saw R., Joshi D., Mandal A. (2023). Formulation and characterization of nanoemulsions stabilized by nonionic surfactant and their application in enhanced oil recovery. Pet. Sci. Technol..

[83] Waheed I., Ali A., Tabassum H., Khatoon N., Lai W.F., Zhou X. (2024). Lipid-based nanoparticles as drug delivery carriers for cancer therapy. Front. Oncol..

[84] Bagheri Karimi M. (2024). Preparation of Lipid Based Nanoparticles from Extracts of Grape Seeds and Evaluation of Their In Vitro Anticancer Effect. Ph.D. Thesis.

[85] Hashim G.M., Shahgolzari M., Hefferon K., Yavari A., Venkataraman S. (2025). Plant-Derived Anticancer Therapeutics and Biopharmaceuticals. Bioengineering.

